# Behavioral Counseling Training for Primary Care Providers: Immersive Virtual Simulation as a Training Tool

**DOI:** 10.3389/fpubh.2019.00116

**Published:** 2019-05-09

**Authors:** Adam G. Gavarkovs

**Affiliations:** Department of Nutrition, Harvard T. H. Chan School of Public Health, Boston, MA, United States

**Keywords:** primary care, behavioral counseling, motivational interviewing, simulation, virtual reality

## Abstract

Behavioral counseling represents an efficacious approach for improving health behaviors on a population level, and the primary care setting is an appropriate context in which to implement this approach. However, evidence suggests that the utilization of behavioral counseling techniques in primary care, including those informed by motivational interviewing, is sub-optimal. Insufficient training has been cited as a barrier to utilizing counseling in the primary care setting. Recent work has evaluated the effectiveness of virtual simulations that can provide access to “virtual” patients while retaining the scalability inherent to a digital medium. However, these educational interventions have been limited to simulations delivered through a two-dimensional screen. More immersive simulations delivered through a head-mounted display can create a realistic practice environment that encompasses a learner's entire field of view, which may confer additional benefits with respect to training outcomes. The purpose of this short article is to briefly review the relevant literature across disciplines to conceptualize the potential effectiveness of this technology as a training tool for behavioral counseling. Immersive virtual simulations are designed to induce a psychological phenomenon referred to as presence, whereby a learner perceives themselves as existing within the virtual environment. As such, immersive virtual simulations can provide opportunities for practice, coaching, and feedback in an environment that closely approximates the clinical setting in which counseling will be delivered. Through its effects on presence, this technology may be particularly useful for developing empathy, which is an important component of counseling. Recommendations for future research are also provided.

## Introduction

In the United States in 2015, the leading causes of mortality included heart disease, cancer, chronic lower respiratory diseases, stroke, and diabetes (1). Contributing to over half of all deaths in the United States, these conditions are potentially preventable through the modification of antecedent, “risky” behaviors (2, 3). As such, finding ways to improve individuals' health-related behaviors on a population level has, and will continue to be, a top public health priority. Behavioral counseling by a trained primary care provider (e.g., physician, nurse practitioner, registered nurse) is one cost-effective method for improving individuals' health behaviors (4, 5). Behavioral counseling may follow the “5A's framework” (assess, advice, agree, assist, arrange), or be informed by principles of Motivational Interviewing (MI), a brief psychotherapeutic method that is designed to promote behavior change through reduced ambivalence and increased motivation for change (6, 7). Systematic reviews and meta-analyses have supported the use of behavioral counseling in primary care as an effective strategy for addressing poor diet, physical inactivity, smoking, substance misuse, and alcohol misuse (8–11). Given physicians' other responsibilities that may limit their time to provide behavioral counseling, it has been recommended that other members of the primary care team, including nurse practitioners, physician assistants, and dietitians, should also be able to provide counseling services (12).

Unfortunately, evidence suggests that the use of behavioral counseling in primary care is not widespread. Among a nationally representative sample of U.S. adults, 32.6% and 33.6% reported receiving advice from their physician about modifying their diet or physical activity, respectively, despite a majority of physicians endorsing behavioral counseling as an important part of their role (13–15). In a systematic review of primary care providers' perceptions of providing counseling for physical activity in the clinical setting, a lack of knowledge/training in counseling was cited as an important barrier by providers (15). Among behavioral counseling curricula included in medical training programs, most involved eight or fewer hours of instructional time, and included primarily didactic and small group instructional methods, with fewer integrating opportunities for practicing with standardized patients (i.e., trained actors) or real patients (16). This is unfortunate given that counseling skills generally decline after initial training and thus must be sustained through periodic training, practice opportunities, coaching, and feedback (17). As a result, studies have reported that a substantial number of primary care providers feel unprepared to provide behavioral counseling in areas such as physical activity, nutrition, childhood obesity, alcohol misuse, and smoking (18–21).

Virtual simulations have received increasing interest as an alternative instructional method for students and primary care providers. These technology-enabled training environments provide learners with the opportunity to interact with a virtual patient to practice behavioral counseling techniques in a realistic, albeit digital, environment. Virtual patients may be controlled algorithmically (i.e., an *agent*) or controlled manually by another human (i.e., an *avatar*) (22). The propensity for learners to apply what they have learned in training, referred to as training transfer, is enhanced when training closely replicates what will occur in practice (23, 24). Patient encounters constructed in a virtual environment may be perceived as equally realistic compared to face-to-face interactions with standardized patients, yet virtual simulations, particularly those that include virtual agents, can be implemented at scale without requiring extensive human resources. Several studies have evaluated the acceptability and effectiveness of virtual simulations for training primary care providers or students how to deliver behavioral counseling in the areas of alcohol misuse, substance misuse, smoking cessation, mental health screening and treatment, and colorectal cancer screening (25–30). The varying methodological quality among this small sample of studies makes it challenging to draw definitive conclusions about the efficacy of virtual simulations, although the randomized trials among this group provide preliminary evidence that virtual simulations may be an effective training strategy for developing some counseling skills among both students and established providers (28, 29). Perhaps the most consistent feature across these studies is that they all evaluate virtual simulations delivered on a two-dimensional computer screen. Learners are typically able to choose what to say through point-and-click or voice selection of a response among a limited “menu” of available responses. However, simulations can also be delivered in immersive virtual environments (IVEs) projected through a head-mounted display (HMD), which affords the opportunity to immerse the learner in a three-dimensional environment that encompasses their entire field of view. In such an environment, learners could interact with a virtual patient through spoken questions (31). Given their immersive nature, these virtual simulations may confer additional benefits to learners that exceed those afforded by simulations delivered through a two-dimensional platform. The purpose of this paper is to integrate the theoretical and applied literature from multiple disciplines to conceptualize the potential effectiveness of immersive virtual simulation as a training tool for behavioral counseling. Findings from a number of studies will be discussed, culminating in a series of research recommendations to empirically test the hypotheses generated in this paper. In a conceptual analysis of MI, Miller and Rose proposed that there are two active components of this counseling approach that result in behavior change (32). The first is a *technical component*, which involves the application of specific strategies to evoke motivation for change. Utilizing strategies associated with other counseling approaches, such as the 5A's framework, could also be considered a part of the technical component of counseling. The second is a *relational component*, which involves a provider's ability to conduct the counseling with empathetic understanding. Evidence suggests both components contribute to positive behavioral outcomes (33). The potential of immersive virtual simulation as a training tool to support the development of each of these components is taken in turn.

## Overview of Immersive Virtual Environments

Immersive virtual environments encompass a number of different technological approaches through which users experience a psychological phenomenon referred to as presence within a technologically-simulated environment (34). Presence has been defined as both a subjective and objective state of consciousness, during which individuals experience a sense of existing within an environment and behave in a way that is consistent with “being in” an environment (35). Co-presence may also occur if an individual perceives themselves as sharing the environment with another individual, such as a virtual patient (36). In 1997, Slater and Sylvia proposed that there are technical features of an IVE system that can affect the propensity of individuals to experience presence, which they call *immersion* (35). A system's level of immersion is a function of several features: *inclusivity*, defined as the extent to which the real environment is shut off from the user's perception; *extensiveness*, defined as the number of sensory modalities (e.g., visual, auditory, haptic) included in the experience; *surrounding*, defined as the holistic nature of the sensory field (as opposed to a narrow field); *vividness*, defined as the quality of the display; *virtual body*, defined as the egocentric (as opposed to exocentric) positioning of the user in the environment; *matching*, defined as the extent to which movement in the virtual environment tracks with a user's actual movements; and *plot*, defined as a temporal unfolding of a sequence of events that are distinct from events occurring in the real world (35). A recent meta-analysis by Cummings and Bailenson found that the tracking level, stereoscopic vision, and field of view in an IVE had particularly strong correlations with presence (37).

## Technical Component of Behavioral Counseling

The technical component of MI is defined as the use of specific MI-consistent (MICO) behaviors to evoke “change talk” from the participant, which subsequently predicts motivation and behavior change (33). Specific MICO behaviors include the use of simple or complex reflective statements and open questions, positive affirmations, offering support, giving advice with permission, raising concern with permission, and emphasizing patient autonomy (33). Other behavioral counseling strategies such as the 5A's could also be considered effective techniques that operate through the “technical” pathway (38). Popular methods for providing realistic experiences in which to learn and apply MICO behaviors include role playing with another learner, using actors as standardized patients, providing expert coaching before and during a simulated clinical encounter, or providing expert feedback after a real clinical encounter (39–42). However, these instructional strategies may be expensive to scale up in education and practice settings, especially considering that repeated opportunities for training with coaching and feedback is recommended to prevent skill decay (17). An expert in MI or other behavioral counseling strategies may not be available in all practice settings to provide ongoing, synchronous performance support. Immersive virtual simulations may be a potentially effective method for learning the technical components of behavioral counseling while retaining the scalability afforded by a digital medium. Last year, Schmid Mast et al. published a paper in Human Resource Development Quarterly, titled *The future of interpersonal skills development: Immersive virtual reality training with virtual humans* (24). As is the case with behavioral counseling training, they report that most interpersonal skills training includes role playing exercises with other trainees or standardized partners, combined with feedback. Schmid Mast and colleagues provide several reasons why immersive virtual simulations may be particularly effective for interpersonal skills development (24). First, training in a virtual space, whether it is in a fully immersive environment with a HMD or a two-dimensional environment on a computer, provides easy access to a practice partner and a coach to provide feedback. This is particularly the case if a virtual patient is algorithmically controlled (i.e., an *agent*) as opposed to controlled manually by a human (i.e., an *avatar*). Given that counseling skills have been shown to decay over time without repeated practice, access to regular training opportunities is critical (17). Second, immersive virtual simulations can create a feeling of co-presence, or the sensation that the learner exists within a world co-occupied by a virtual patient (36), while still maintaining a level of artificiality that connotates a risk-free environment. For example, research suggests that immersive simulations of a presentation may reduce anxiety among those with a fear of public speaking (43). For behavioral counseling training, this balance may be better struck in virtual simulations compared to role-playing with colleagues or a face-to-face simulated patient, which may be perceived as less authentic. Third, with the aid of algorithmic behavioral control, learners can practice interacting with a number of unique virtual agents. These could include patients who are struggling with different behavioral challenges (e.g., physical inactivity, risky sexual practices) and who are more or less amenable to change. Training transfer is enhanced when training experiences can better replicate what will occur in practice, so this may better prepare students or providers for the dynamic and adaptive nature of patient-provider interactions (24). Engaging in training within an authentic, immersive environment may also impact providers' attitudes about the efficacy of behavioral counseling. Several studies have found that behavioral counseling curricula in residency programs can improve knowledge and self-efficacy but not attitudes toward counseling (44, 45). This is unfortunate given that the perceived efficacy of behavioral counseling is a significant predictor of providers' provision of counseling during an appointment (46). If a learner feels strong presence within a virtual simulation, it may be that experiencing positive counseling outcomes within this environment influences the perceived efficacy of counseling more strongly than face-to-face interactions with standardized patients or other learners.

## Relational Component of Behavioral Counseling

Empathetic understanding in the context of the patient-provider relationship has been defined as “the ability of physicians to imagine that they are the patient who has come to them for help” (p. 367) (47), and has been linked with better counseling outcomes (48). Achieving empathetic understanding among providers may be challenging; there are a significant number of care providers who hold weight or addiction-related biases (49, 50). One way to develop empathic understanding is by promoting *perspective taking*, which is an exercise in trying to see the world from someone else's perspective (51). Research has shown that perspective-taking results in an increased willingness to provide help for another (52). Role playing with another medical student, resident, or provider may be a useful way of practicing perspective taking, as learners take turns playing the patient, requiring the construction of an alternative “patient” perspective (53). Although having a character prompt (i.e., play a 65-year-old who has been living with diabetes for 20 years) may enhance learners' ability to take on the perspective of a patient, this process requires significant cognitive effort, impeded by the fact that the learner remains psychologically present in the “reality” in which they are a provider or student. Studies have found that individuals are less able to take the perspective of others when they are under high cognitive load, illustrating the cognitively demanding nature of this process (54). Furthermore, some individuals may be more or less able or motivated to engage in this activity (55). Immersive virtual simulations may enhance the ability of individuals to take the perspective of others, as it can help facilitate the construction of an alternative perspective without requiring users to expend cognitive effort to create this mental model from their imagination (56). In a study from 2006, Yee and Bailenson investigated the effects of IVE-mediated perspective taking on negative stereotyping of older adults (57). Participants were immersed in a virtual environment, having been randomized to embody an avatar of an elderly person or a younger person of the same gender, during which time they completed a variety of tasks designed to enhance their perceived embodiment of the young or old avatar. After the immersive experience, compared to participants randomized to the young avatar, those randomized to the old avatar associated the elderly with significantly more positive traits in a word association test. Ahn et al. (58) directly compared methods for perspective taking by randomizing participants to an embodied experience condition in which the participant was immersed in a virtual environment that simulated red-green colorblindness or a perspective taking condition in which the participant was immersed in a virtual environment that had normal coloring but was asked to imagine being colorblind (58). The researchers found that, particularly among those with low perspective-taking ability, those in the embodied experience condition reported higher self-other overlap (i.e., a feeling of connection with another person), more favorable attitudes toward those with colorblindness, and voluntarily spent more time helping a person with colorblindness after the experiment. Furthermore, a higher sense of realism during the embodied experience predicted stronger self-other overlap. The results of these studies suggest that perspective taking within an IVE may make it easier for providers to lose a feeling of self-awareness and cognitively “embody” the perspective of their patients. Because this perspective is externally generated (i.e., it is not being constructed from the user's mind), it is not affected by pre-existing weight- or addiction-related biases that influence their established mental schemas of certain patients. If students or primary care providers were to embody a patient struggling with obesity or substance misuse and experience some aspects of their life related to their condition through the eyes of the patient, they may develop a more empathetic understanding toward their vulnerable patients. Providers could also benefit from seeing a negative or stigmatizing clinical experience from the patient's perspective. Given the explicit and implicit weight biases among providers, it could be that many are using inappropriate, hurtful, judgmental, or accusatory language, either purposefully or unintentionally (49). By viewing a negative encounter with a virtual provider from the patient's perspective, providers may develop more empathy for their patients.

## Discussion

The purpose of this multi-disciplinary review was to conceptualize the potential effectiveness of immersive virtual simulation as a tool for training primary care providers how to deliver behavioral counseling in their practice. Its demonstrated effects on interpersonal skills development and empathic understanding cover the technical and relational components of behavioral counseling. Although studies of virtual simulations delivered through a two-dimensional computer screen have reported positive effects on counseling skills, and preliminary evidence suggests this may translate to counseling utilization in practice, no studies to date have extended this work to investigate the effects of immersive virtual simulations delivered through a HMD (25–30). Future trials should be undertaken to evaluate the effectiveness of this innovative technology with respect to a variety of behavioral counseling topics, such as smoking cessation, substance misuse, physical activity, and dietary modification, among others. Furthermore, the opportunity exists to evaluate other forms of technology-mediated simulation that may be more immersive than those delivered on a computer screen, such as life-size projections of virtual patients (59, 60). Lessons from studies evaluating virtual simulations delivered through two-dimensional screens may inform the rigorous design of such trials. First, studies should include expert-rated measures of counseling effectiveness, such as the Motivational Interviewing Treatment Integrity Code (61), as opposed to self-reported increases in skill. Second, randomized controlled trials may consider including an active comparison group to compare the effectiveness of immersive virtual simulation against more typical training methods such as in-person role playing or face-to-face standardized patient interactions. Such studies would provide more persuasive evidence to support the adoption of this technology compared to trials that included an inactive control group. Other comparative effectiveness trials could assess the effectiveness of immersive virtual simulations with different features (e.g., different degrees of freedom of tracking, voice vs. point-and-click selection) or compare blended approaches (e.g., an online webinar and virtual simulation compared to only a webinar) to assess whether immersive virtual simulations are more effective when combined with other scalable training strategies. Third, studies should provide participants with the opportunity to immerse themselves in the virtual simulation multiple times over an extended period of time and include more long-term assessments to determine the effects on both skill acquisition and retainment. Periodic retraining may promote the retention of counseling skills and immersive virtual simulations provide the opportunity for regular access at low cost. Finally, studies should evaluate the barriers to adoption and use of immersive virtual simulations. Although they may offer additional benefits compared to virtual simulations delivered on a two-dimensional screen, the technical requirements for immersive virtual simulations (e.g., a head-mounted display, a mobile phone or computer with the necessary hardware) may present logistical and financial barriers to its uptake. Immersive virtual simulations may represent a potentially effective training tool over and above existing methods for training primary care providers; future research needs to be undertaken to empirically test this claim.

## Data Availability

No datasets were generated or analyzed for this study.

## Author Contributions

The author confirms being the sole contributor of this work and has approved it for publication.

### Conflict of Interest Statement

The author declares that the research was conducted in the absence of any commercial or financial relationships that could be construed as a potential conflict of interest.
